# Traumatic Brain Injury, Microglia, and Beta Amyloid

**DOI:** 10.1155/2012/608732

**Published:** 2012-05-14

**Authors:** Rebekah C. Mannix, Michael J. Whalen

**Affiliations:** ^1^Division of Emergency Medicine, Department of Medicine, Children's Hospital Boston, Harvard Medical School, Boston, MA 02124, USA; ^2^Neuroscience Center, Massachusetts General Hospital, Harvard Medical School, Charlestown, MA 02129, USA; ^3^Department of Pediatrics, Massachusetts General Hospital, Harvard Medical School, Charlestown, MA 02129, USA

## Abstract

Recently, there has been growing interest in the association between traumatic brain injury (TBI) and Alzheimer's Disease (AD). TBI and AD share many pathologic features including chronic inflammation and the accumulation of beta amyloid (A**β**). Data from both AD and TBI studies suggest that microglia play a central role in A**β** accumulation after TBI. This paper focuses on the current research on the role of microglia response to A**β** after TBI.

## 1. Introduction

Recently, there has been growing interest in the association between traumatic brain injury (TBI) and Alzheimer's Disease (AD). The interest grew from several lines of evidence, including epidemiological studies that demonstrated an association of TBI and the development of AD later in life [1–7] and autopsy studies that showed acute and chronic AD-like pathology in TBI victims [8, 9]. While most of the studies investigating the association of AD and TBI have focused on the accumulation and clearance amyloid- *β* (A*β*) [2, 8, 9], chronic neuroinflammation is also a common feature of AD and TBI, and microglia likely play a central role [10, 11]. In AD, microglia are recruited to newly formed A*β* plaques, where microglial activation functions as a double-edged sword, promoting beneficial responses such as A*β* clearance [12–14] while also eliciting a proinflammatory response [12]. Similar patterns of microglia activation have been demonstrated both acutely and chronically after TBI [15, 16]. 

This paper will explore the current research on the role of microglia response to A*β* after TBI. Although there are few studies that directly examine microglial reaction to trauma-induced A*β*, data from TBI and AD experimental and human studies will be used to make an argument for a central role of microglia in acute and chronic responses to A*β*-mediated secondary injury after TBI.

## 2. General Microglial Response after TBI

TBI is a disease process in which mechanical injury initiates cellular and biochemical changes that perpetuate neuronal injury and death over time, a process known as secondary injury. Secondary injury begins minutes after injury and can continue years after the initial insult. Mechanisms implicated in secondary injury after TBI include glutamate excitotoxicity, blood-brain barrier disruption, secondary hemorrhage, ischemia, mitochondrial dysfunction, apoptotic and necrotic cell death, and inflammation [17].

As the primary mediators of the brain's innate immune response to infection, injury, and disease, microglia react to injury within minutes. In fact, microglia may represent the first line of defense following injury [18]. Microglial activation has been demonstrated as early as 72 hours after injury in human TBI victims and can persist for years after injury [15, 16, 19, 20]. Experimental models have recapitulated these findings, with chronic microglial activation being demonstrated weeks to months after injury [21–24].

Gene-profiling studies also strongly implicate early microglial activation after TBI. 

Markers of microglial activation (CD68, MHC-II), stress responses (p22phox, heme oxygenase 1), and chemokine expression (CXCL10, CXCL6) have been shown to markedly increase after experimental models of TBI [25]. Consistent with early microglial activation after injury, experimental models have demonstrated rapid increases in expression of both IL-1*β* and TNF-*α* after injury [26, 27]. Much of this proinflammatory cascade may be mediated by Il-1IR, which is strongly expressed on microglia [27–29]. In addition to proinflammatory cytokines, activated microglia also produce other neurotoxic products after injury such as nitric oxide (NO) and superoxide free radicals that generate reactive oxygen species (ROS) and reactive nitrogen species (RNS).

Microglia also produce a number of neuroprotective substances after injury, including anti-inflammatory cytokines (IL-10, IL-1 receptor antagonist (Il-1ra)) and neurotrophic factors (nerve growth factor, transforming growth factor *β* (TGF-*β*). IL-10, which is elevated acutely after injury in humans [30], has been shown to have beneficial effects in experimental models of injury [31]).

These neuroprotective effects may be a result of suppressed microglial production of proinflammatory cytokines [31, 32]. TGF-*β* also has also been shown to have neuroprotective effects after injury, including improved function, decreased lesion size, and decreased iNOS production [33, 34].

## 3. Microglial Response to A*β* after TBI

A*β*, which is elevated acutely after TBI, may be a key mediator of microglial activation in this setting [35]. In autopsy studies, A*β* plaques, a hallmark of AD, were present in as many as 30% of TBI victims (including children) [8, 9]. The plaques found in TBI patients, which are strikingly similar to those observed in the early stages of AD, develop rapidly and can appear within a few hours after injury [9, 36]. TBI-induced increases in A*β* have been successfully replicated in animal models of brain trauma [37–40]. Moreover, A*β* accumulation after TBI has also been shown to be associated with increases in the enzymes necessary for A*β*-genesis, including BACE1 protein (*β*-secretase) and the gamma secretase complex proteins [41–43].

**Figure 1:**
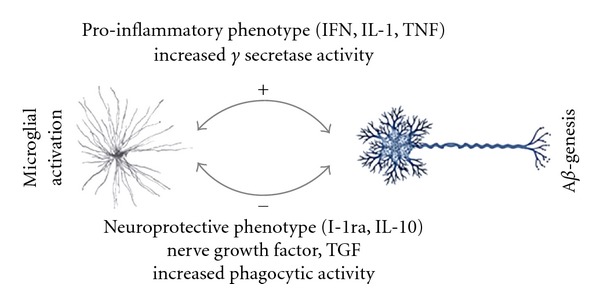
Schematic depiction of the beneficial and detrimental effects of the interaction A*β* -genesis and microglia after traumatic brain injury.

Microglia may have a dual role in A*β* accumulation and clearance (Figure 1). Following closed head injury, microglia have been shown to have increased expression of the gamma secretase complex proteins, suggesting a role for microglia in posttraumatic A*β*-genesis [43]. Polymorphisms in the A*β*-degrading enzyme neprilysin have been shown to affect rates of A*β* accumulation after TBI [2], suggesting the possibility that changes in microglial neprilysin expression may be a pathologic mechanism in post-TBI A*β* accumulation in addition to the known relevance to AD [12]. Moreover, proinflammatory cytokines expressed by microglia, including interferon-*γ*, interleukin-1*β*, and tumor necrosis factor-*α*, can specifically stimulate gamma-secretase activity, concomitant with increased production of A*β* and the intracellular domain of APP (AICD) [44]. Further evidence suggests that increases in the numbers of neurons with elevated ß-APP concentrations after TBI correlate with increases in the number of activated microglia expressing IL-1*α* and that clusters of dystrophic neurites containing ß-APP are nearly universally associated with activated microglia expressing IL-1*α* [45]. Microglia containing A*β* have also been described in association with TBI-induced A*β* plaques, suggesting that phagocytic clearance of plaques may occur [46]. 

In addition to the temporospatial relationship of microglial and A*β* after TBI, much of the evidence regarding the important role of microglia in the modulation of TBI-induced A*β* is indirect. Most of this indirect evidence is derived from drug studies, which, while not designed to demonstrate the important interaction of microglial with TBI-induced A*β*, have served to demonstrate this vital interaction. Many of the studies that have indirectly elucidated the interaction of microglia and A*β* after TBI have focused on therapeutics targeting postinjury inflammation. Minocycline, a compound whose anti-inflammatory properties (including attenuating microglial activation) have been widely demonstrated in different models of TBI [47, 48], has also been shown to preclude formation of A*β* through restoration of the nonamyloidogenic *α*-secretase pathway of APP processing [49]. Interestingly, it has been shown that some other anti-inflammatory compounds that exert neuroprotective effects in TBI, such as nonsteroidal anti-inflammatory drugs, cholesterol-lowering drugs, and steroid hormones, also enhance the *α*-secretase pathway [50–53]. It is unclear from these studies whether the anti-inflammatory and pro*α*-secretase effects are merely parallel processes, although certainly diversion of APP from the production of A*β*, which has known proinflammatory properties including stimulation of microglial activation, may itself adequately explain both the anti inflammatory and anti amyloidogentic effects.

Additional studies support the observation that suppression of microglial activation after TBI is also associated with decreases in injury-induced A*β*. Apoe mimetic peptides, liver X-receptor (LXR) agonists, and 3-Hydroxy-3-methylglutaryl coenzyme A reductase inhibitors (statins) have all been shown to attenuate microglial activation and mitigate increases in A*β* after TBI [54–56]. It should be noted, however that none of these earlier studies can definitively lead one to conclude whether A*β* is a cause, product, or mere marker of microglial activation and secondary injury after TBI. However, what can be concluded is that A*β* is an excellent indicator of microglial activation following TBI.

## 4. Conclusions and Future Directions

The link between trauma, microglial activation, and A*β* is likely to be extremely complex, and work in this field remains in its infancy. Much of the work is derived from studies in AD, though the time course and long-term sequelae of TBI and AD may require separate lines of investigation. One recurring issue is the role of A*β* in the pathogenesis of microglial activation after brain injury which parallels the increasing emphasis on microglial function in the pathogenesis of AD.

Prior studies in AD suggest that microglial clearance of A*β* declines with aging [12]. It is therefore important to understand how TBI-induced A*β* alters long-term microglial function and A*β*-clearance. Future work should focus on whether blocking A*β*-genesis after TBI alters short- and long-term microglial activation. In addition, therapeutics targeting microglial-mediated A*β* clearance, such as the new AD therapeutic bexarotene [57], may hold promise as new modalities to treat TBI patients.

In this paper, we have attempted to show how a mechanistic understanding of the interaction of A*β* and microglia after TBI could have significant implications for therapeutics, especially for those at the highest risk for TBI, including those in the military and those who engage in contact sports. Furthermore, the advancement of drug discoveries in the field of TBI, such as sex steroids or Apoe mimetics which alter both microglial function and A*β* metabolism, may have potentially important roles in TBI as well as other neurodegenerative diseases. Finally, as we advance our mechanistic understanding of the interaction between microglia and A*β* after TBI, it is important to understand whether therapeutic interventions targeting microglia and A*β* will have any effect on long-term cognitive sequelae in TBI victims.
